# Dihydroartemisinin Regulates the Th/Treg Balance by Inducing Activated CD4+ T cell Apoptosis via Heme Oxygenase-1 Induction in Mouse Models of Inflammatory Bowel Disease

**DOI:** 10.3390/molecules24132475

**Published:** 2019-07-05

**Authors:** Si Chao Yan, Ya Jie Wang, Yu Jie Li, Wei Yan Cai, Xiao Gang Weng, Qi Li, Ying Chen, Qing Yang, Xiao Xin Zhu

**Affiliations:** 1 Artemisinin Research Center, China Academy of Chinese Medical Sciences, Beijing 100700, China; 2 Institute of Chinese Materia Medica, China Academy of Chinese Medical Sciences, Beijing 100700, China

**Keywords:** dihydroartemisinin, CD4+ T subsets, heme oxygenase-1, ulcerative colitis, Crohn’s disease

## Abstract

Dihydroartemisinin (DHA) is a derivative of the herb *Artemisia annua L.* that has prominent immunomodulatory activity; however, its underlying mechanism remains elusive. Inflammatory bowel disease (IBD) is an idiopathic inflammatory condition characterized as an autoimmune disorder that includes dysfunctions in the T helper (Th)/T regulatory cell (Treg) balance, which normally plays pivotal roles in immune homeostasis. The aim of this study was to explore the potential of DHA to ameliorate IBD by restoring the Th/Treg cell balance. To this end, we established mouse models of colitis induced by oxazolone (OXA) and 2,4,6-trinitro-benzene sulfonic acid (TNBS). We then treated mice with DHA at 4, 8, or 16 mg/kg/day. DHA treatment ameliorated colitis signs and reduced lymphocyte infiltration and tissue fibrosis. Moreover, DHA decreased the numbers of Th1 and Th17 cells and Th9 and Th22 cells in TNBS- or OXA-induced colitis, respectively, and increased Tregs in both models. DHA (0.8 mg/mL) also inhibited activated CD4+ T lymphocytes, which was accompanied by apoptosis induction. Moreover, it promoted heme oxygenase-1 (HO-1) production in vitro and in vivo, concomitant with CD4+ T cell apoptosis and restoration of the Th/Treg balance, and these effects were blocked by treatment with the HO-1 inhibitor Sn-protoporphyrin IX. Overall, these results suggest that DHA is a novel and valuable candidate for IBD therapy or Th/Treg immunoregulation.

## 1. Introduction

Inflammatory bowel disease (IBD), comprising ulcerative colitis (UC) and Crohn’s disease (CD), is an idiopathic autoimmune disease characterized by non-specific, relapsing, and precarious inflammatory reactions in the gastrointestinal tract. In addition to repeated inflammation, fibrosis is another major contributor to the pathogenic process [1]. The main symptoms of IBD include weight loss, diarrhea, hematochezia, abdominal pain. Moreover, the morbidity of IBD has been increasing annually in various regions, resulting in substantial medical expenses [2]. Patients with IBD also have an increased risk of other diseases such as anemia [3], acute arterial events [4], and colorectal cancer [5]. The mainstays of IBD treatment include dietary, surgical, and medical approaches [6]; however, many patients are still reluctant to receive treatment due to the associated high costs, toxic effects, and long-term uncertainty [7]. New alternative medical strategies for IBD are thus desperately needed. As long-term medication is necessary for IBD therapy, dihydroartemisinin (DHA), a derivative of the artemisinin family, which has been used for clinical malaria treatment for decades and is associated with high efficacy, minimal side effects and low cost, could be seen as a promising candidate.

Artemisinins comprise a group of sesquiterpene trioxane lactones extracted from the Chinese herb *Artemisia annua L.* Professor Tu Youyou was awarded the Lasker Award as well as a Nobel Prize for Physiology and Medicine based on her discovery of the artemisinin family and its potential for malaria treatment [8,9]. In particular, DHA is an artemisinin derivative that was shown to act as a potent immunomodulator; further, it was initially demonstrated to be an effective treatment option for SLE by Dr. Tu Youyou’s research team and is currently being assessed in a Chinese clinical trial. DHA and its derivatives were also reported as candidates for the treatment of some other autoimmune diseases like experimental autoimmune encephalomyelitis (EAE) [10] and collagen-induced arthritis [11].

IBD is also a type of autoimmune disease. Although the precise pathogenesis remains elusive, disorders in CD (Cluster of differentiation) 4+ T cell subsets are perceived as fundamental to its pathology [12]. CD4+ T cells are abundant in the lamina propria and are activated upon contact with food and microbial antigens in the gastrointestinal tract [13,14]. Among CD4+ T cell subsets, disease progression is considered to be strongly linked to disorders in the balance between T helper (Th) and T regulatory cell (Tregs) [15], essential lineages of CD4+ T cell subsets. The aberrant immune responses triggered by Th cells like Th1, Th17 [16], Th9 [17], and Th22 [18] cells play critical roles during the pathogenic process. As an opposing mechanism, Treg cells can attenuate the aberrant immune response against self-antigens to induce immunological self-tolerance [19]. As new information is continuously revealed about the reciprocal regulation of Th and Treg cells, new concepts and strategies for immune dysfunction intervention are rapidly emerging.

For example, heme oxygenase-1 (HO-1) has been reported to contribute to immune suppression by regulating CD4+ T cell subsets. HO-1 is a rate-limiting enzyme involved in the process of heme metabolism [20] and functions by catalyzing heme to carbon monoxide, iron, and biliverdin in response to oxidative stress [21], resulting in a wide range of anti-inflammatory and anti-oxidant effects. One proposed mechanism underlying these effects involves the suppression of CD4+ T cell activation via the regulation of metabolic products catalyzed by HO-1 on mature dendritic cells. Alternatively, HO-1 could affect some lineage-related cytokines and transcription factors to participate in the Th/Treg-mediated immune response [22].

Given that the heme metabolic process was proven to be the trigger and target of the artemisinin family [23], and that HO-1 can regulate Th/Treg disorders (which comprise the pathogenic basis of IBD), we hypothesized that DHA would have protective effects on IBD, and that the underlying mechanism would comprise Th/Treg modulation through the regulation of HO-1. To test this hypothesis, we established mouse models of colitis induced by the intracolonic application of oxazolone (OXA) and 2,4,6-trinitro-benzene sulfonic acid (TNBS), which have histological features that resemble human UC and CD, respectively [24]; this was followed by treatment with or without DHA. The effect of DHA treatment on colitis sign alleviation in vivo was assessed based on weight loss, disease signs, survival rate, histopathological observations, Th/Treg balance-related factors, and HO-1 in lamina propria mononuclear cells (LPMCs). In particular, previous studies demonstrated that Th and Treg cells play important roles during the development of IBD, and an OXA model has been used for studies on Th9 cells [17], whereas a TNBS model has been used to assess Th1 and Th17 cells [23]. Accordingly, we used OXA-induced colitis to study the effect of DHA on Th9 and potentially Th22 cells and TNBS-induced colitis for Th1 and Th17 cells. The specific role of HO-1 with respect to the underlying mechanism of DHA was further examined using the HO-1 inhibitor Sn-protoporphyrin IX (SnPP).

## 2. Results

### 2.1. DHA Ameliorates OXA-Induced Colitis in a Dose-Dependent Manner in Vivo

**Figure 1:**
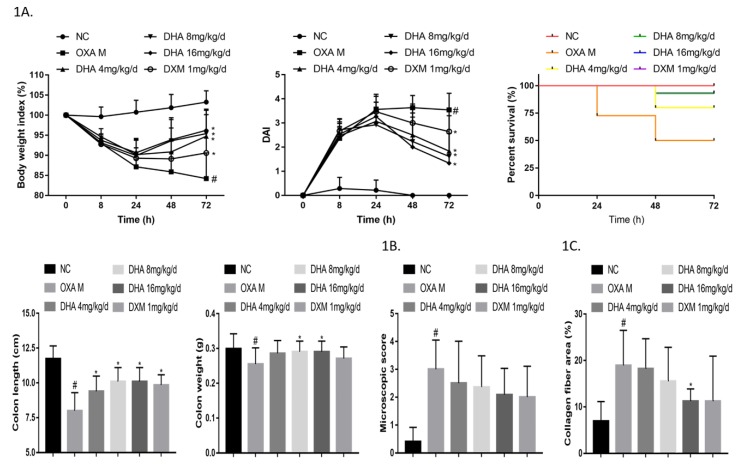
Dihydroartemisinin (DHA) ameliorates oxazolone (OXA)-induced colitis in a dose-dependent manner in vivo. (**1A**) Inflammatory bowel disease (IBD) was elicited in mice via OXA intracolonic application. Body weight index, disease activity index (DAI) score, mortality, colon weight, and colon length of each group are shown (*n* = 15–22). (**1B**) Microscopic scores of colon H&E-stained images in each group are shown (*n* = 8–10). (**1C**) Percent collagen fiber areas based on images of Sirius Red-stained colons in each group are shown (*n* = 8–10). (Compared to the NC group, #*p* <0.05, compared to the model group, *p <0.05).

Both OXA and TNBS induced severe colitis in mice, as evidenced by weight loss, diarrhea, and bloody stools. As shown in Figure 1A, OXA-administered mice suffered the most serious disease signs and had the highest mortality rates. However, DHA treatment reversed the weight loss and decreased disease activity index (DAI) scores and mortality rates in a dose-dependent manner. In particular, OXA-induced mice treated with 16 mg/kg DHA per day were all alive at the end of the experimental period, similar to that observed for mice in the negative control group. The colons removed from OXA-treated mice were markedly shortened and lighter, and the protective effect of DHA was observed in all treatment groups. Compared to those in OXA mice, H&E (Hematoxylin-Eosin)-stained colonic sections of DHA-treated mice showed reduced leukocyte infiltration and structural destruction (Figure 1B and Figure S1A), whereas Sirius-Red staining revealed a lower degree of tissue fibrosis (Figure 1C and Figure S1B). These results indicated that DHA could ameliorate OXA-induced colitis in mice in a dose-dependent manner in vivo.

### 2.2. DHA Ameliorates TNBS-Induced Colitis in a Dose-Dependent Manner in Vivo

**Figure 2:**
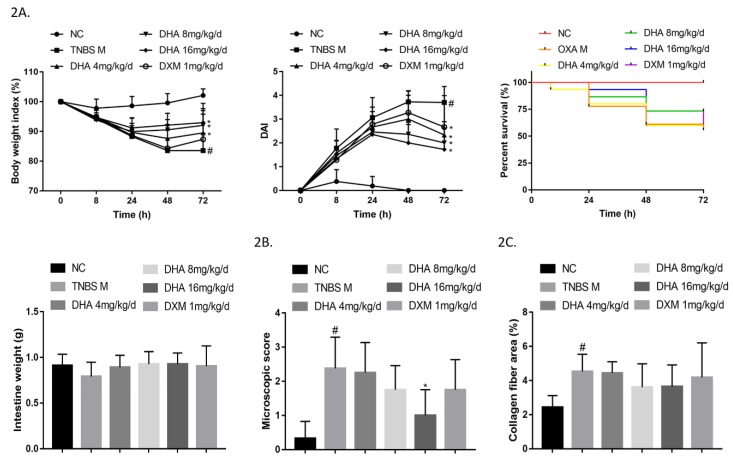
Dihydroartemisinin (DHA) ameliorates 2,4,6-trinitro-benzene sulfonic acid (TNBS)-induced colitis in a dose-dependent manner in vivo. (**2A**) Inflammatory bowel disease (IBD) was elicited in mice via TNBS intracolonic application. Body weight index, disease activity index (DAI) score, mortality, and intestine weight of each group are shown (*n* = 15–18). (**2B)** Microscopic scores of colon H&E-stained images in each group are shown (*n* = 7–9). (**2C**) Percent collagen fiber areas based on images of Sirius Red stained intestines in each group are shown (*n* = 7–9) (Cmpared to the NC group, #*p* <0.05, compared to the model group, *p <0.05).

Similar to that observed for OXA-induced colitis, DHA improved weight loss, disease activity index (DAI) scores, and mortality rates in TNBS-induced colitis. Although there was no significant difference, DHA tended to increase intestine weight (Figure 2A) and improve leukocyte infiltration and tissue fibrosis based on H&E (Figure 2B and Figure S2A) and Sirius-Red (Figure 2C and Figure S2B) staining. These results indicated that DHA could ameliorate TNBS-induced colitis in mice in a dose-dependent manner in vivo.

### 2.3. DHA Regulates the Th/Treg Balance in OXA- and TNBS-Induced Colitis

Quantitative RT-PCR showed that DHA significantly reduced expression levels of the transcription factors *PU.1* (Th9) and *AHR* (Th22) and increased levels of *Foxp3* (Treg) in the OXA colitis model. Moreover, DHA significantly decreased expression levels of mRNA encoding the cytokines IL (Interleukin) -9 and IL-22, whereas it tended to increase IL-10 expression, albeit without a statistically significant difference (Figure S3A). In addition, DHA inhibited the expression of RORγt (Retinoid-related orphan receptorγt) (Th17) and enhanced the expression of Foxp3 (Treg) in the TNBS colitis model, and 4 mg/kg DHA per day increased IL-10 expression levels significantly; however, DHA had no obvious effect on the expression levels of T-bet (Th1) and IFNγ (Interferronγ) (Figure S3B).

**Figure 3:**
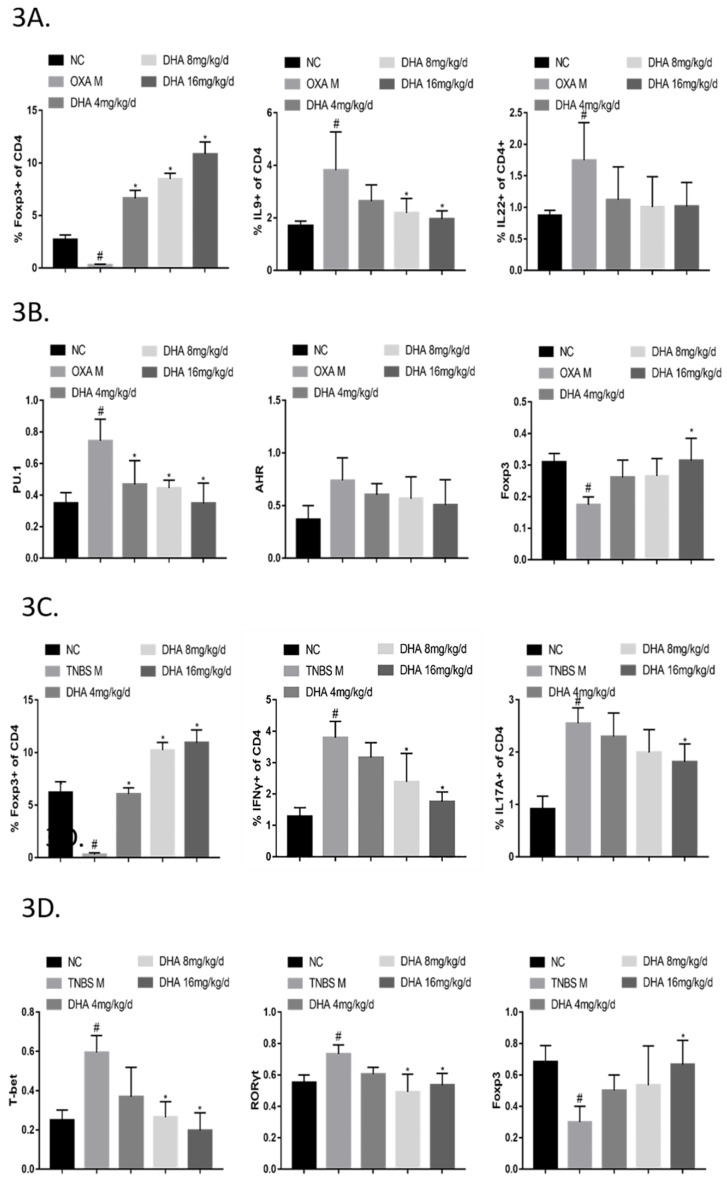
Dihydroartemisinin (DHA) regulates the Th/Treg balance in oxazolone (OXA)- and 2,4,6-trinitro-benzene sulfonic acid (TNBS)-induced colitis. (**3A**) Lymphocytes were isolated from mouse spleens. CD4+ IL (Interleukin) 9+, CD4+IL22+ and CD4+CD25+Foxp3+ cells were determined by flow cytometry. (*n* = 5). (**3B**) Lamina propria mononuclear cells (LPMCs) were isolated and subjected to RIPA lysis for protein collection. The protein amounts of PU.1 (Spi-1 proto-oncogene), AHR (Aryl hydrocarbon receptor), Foxp3 (Forkhead box P3) were estimated by capillary electrophoresis immunoblotting. (*n* = 3). (**3C**) Lymphocytes were isolated from mouse spleens. CD4+IFNγ (Interferronγ) +, CD4+IL (Interleukin) 17A+ and CD4+CD25+Foxp3+ cells were determined by flow cytometry. (*n* = 5). (**3D**) Lamina propria mononuclear cells (LPMCs) were isolated and subjected to RIPA lysis for protein collection. The protein amounts of T-bet (T-box expressed in T cells), Foxp3, and HO-1 were estimated by capillary electrophoresis immunoblotting. (n = 3). (Compared to NC group, #*p* <0.05, compared to model group, *p <0.05).

To further explore the regulatory effect of DHA on CD4+ T cell subsets, flow cytometry was conducted on the spleen lymphocytes of mice in each group. In the OXA colitis model, DHA markedly inhibited the numbers of Th9 cells and increased the numbers of Treg cells. DHA also showed an inhibitory effect on Th22 cells, although the difference was not statistically significant (Figure 3A). In the TNBS colitis model, DHA significantly decreased the numbers of Th1 and Th17 cells and increased Treg cell numbers (Figure 3C).

Moreover, DHA significantly decreased the protein expression levels of PU.1 (Spi-1 proto-oncogene) and AHR (Aryl hydrocarbon receptor) in the LPMCs of the OXA colitis model (Figure 3B) and decreased the protein levels of T-bet and RORγt in the TNBS colitis model (Figure 3D). DHA also significantly increased Foxp3 protein expression in both models (Figure 3B,D).

### 2.4. DHA Suppresses Activated CD4+ T Cell Subsets by Inducing Apoptosis via HO-1

**Figure 4:**
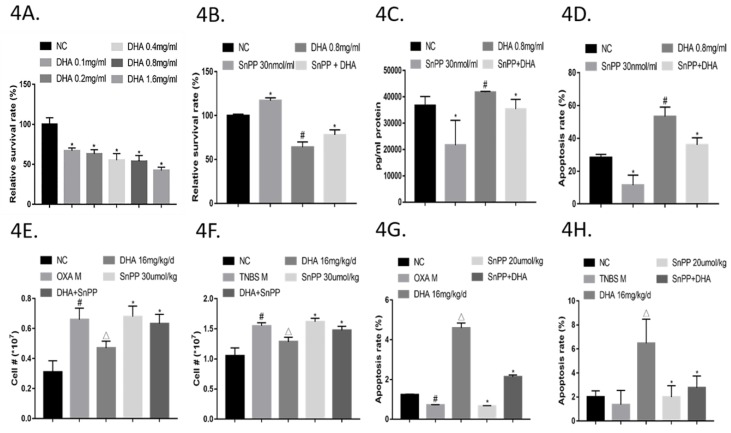
Dihydroartemisinin (DHA) suppresses activated CD4+ T cell subsets by inducing apoptosis via HO-1. (**4A**) Purified spleen CD4+ T cells were activated with plate bound anti-CD (Cluster of differentiation) 3 and anti-CD28, with DMSO (Dimethyl sulphoxide) or DHA, in a concentration-dependent manner. Surviving cell ratios were monitored after 72 h of culture. (*n* = 3). (**4B**) Purified CD4 + T cells were activated with plate bound anti-CD3 and anti-CD28, with DMSO, Sn-protoporphyrin IX (SnPP), DHA, or SnPP+DHA. Surviving cell ratios were monitored after 72 h of culture. (*n* = 3). (**4C**) As described in (**4B**), cell culture supernatant of the four groups was collected separately to evaluate HO-1 protein levels by ELISA (*n* = 3). (**4D**) As described in (**4B**), remaining cells were stained with Annexin-V and propidium iodide (PI). The percentages of each population were detected by flow cytometry. (*n* = 3). (**4E**) Lymphocytes from colon lamina propria samples were collected from NC, OXA, DHA (16 mg/kg/d), SnPP (30 µM/kg), and DHA + SnPP groups. Cell numbers were calculated. (*n* = 3). (**4F**) Lymphocytes from intestine lamina propria samples were collected from the NC, OXA, DHA (16 mg/kg/d), SnPP (30 µM/kg), and DHA + SnPP groups. Cell numbers were calculated. (*n* = 3). (**4G)**. Colons removed from NC and OXA mice were stained with TUNEL. The percentages of positive cells were calculated in each group. (*n* = 3). (**4H**) Intestines from NC and TNBS mice were stained with TUNEL. The percentages of positive cells were calculated in each group. (*n* = 3). (Compared to the NC group, #*p* <0.05, compared to the model group, △*p* <0.05, compared to DHA group, *p <0.05).

As shown in Figure 4A, DHA (0.1–1.6 mg/mL) markedly reduced the ratio of surviving CD4+ T cells upon anti-CD3/CD28 stimulation in a concentration-dependent manner. Notably, the suppressive effect of DHA (0.8 mg/mL) was partially reversed by treatment with the HO-1 inhibitor SnPP (30 nM) (Figure 4B). Moreover, DHA induced apoptosis in activated CD4+ T cells according to a propidium iodide (PI) double staining assay, which was also partly reversed by SnPP (Figure 4D and Figure S4A). Consistently, HO-1 protein levels in the cell culture supernatant were increased and decreased by DHA and SnPP, respectively, and HO-1 upregulation was significantly neutralized by SnPP (Figure 4C).

Further, LPMCs were harvested from OXA and TNBS colitis model groups; cell numbers were significantly decreased by DHA, which was reversed by SnPP (Figure 4E,F). In addition, the percentages of TUNEL (Terminal deoxynucleotidyl transferase-mediated dUTP-biotin nick end labeling assay)-positive cells in the DHA-treated groups were higher than those in the OXA and TNBS colitis model groups. However, treating OXA or TNBS-induced colitis model mice with SnPP+DHA significantly decreased the numbers of TUNEL-positive cells (Figure 4G,H, Figure S4B and S4C). These results suggested that DHA induces activated CD4+ T cell apoptosis by regulating HO-1 expression and production.

### 2.5. DHA Ameliorates OXA- and TNBS-Induced Colitis via HO-1

**Figure 5:**
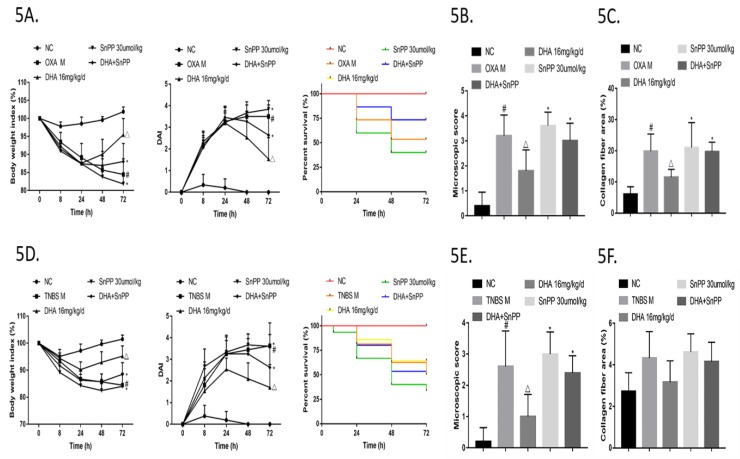
Dihydroartemisinin (DHA) ameliorates oxazolone (OXA)- and 2,4,6-trinitro-benzene sulfonic acid (TNBS)-induced colitis via HO-1. (**5A**) Inflammatory bowel disease (IBD) was elicited in mice via OXA intracolonic application. Body weight index, disease activity index (DAI) score and mortality rate of each group are shown (*n* = 14–16). (**5B**) Mice were administered OXA. Microscopic scores of colon H&E-stained images in each group are shown (*n* = 5–6). (**5C**) Mice were administered OXA. Percent collagen fiber areas based on images of Sirius Red-stained colons in each group are shown (*n* = 8–10). (**5D**) IBD was elicited in mice via TNBS intracolonic application. Body weight index, DAI score, and mortality rate of each group are shown (*n* = 14–16). (**5E**) Mice were administered TNBS. Microscopic scores of intestine H&E-stained images in each group are shown (*n* = 5–6). (**5F**) Mice were administered TNBS. Percent collagen fiber areas based on images of Sirius Red-stained intestines in each group are shown (*n* = 4–6). (Compared to NC group, #*p* <0.05, compared to model group, △*p* <0.05, compared to DHA group, *p <0.05).

Next, mice with OXA-induced colitis were treated with SnPP+DHA, which resulted in greater weight loss, more serious disease signs, and higher mortality compared to those in animals treated with DHA alone (Figure 5A). H&E staining of histopathological sections of the colon showed greater lymphocyte infiltration in the SnPP + DHA group than in the DHA treated group. (Figure 5B and Figure S5A). In addition, the area of collagen fibrosis in the colon was larger in the SnPP + DHA group according to Sirius-Red staining (Figure 5C and Figure S5B). Similarly, in the TNBS colitis model, disease signs in the SnPP + DHA treatment group were much worse, and mortality was higher than those in the DHA-treated group (Figure 5D). A greater degree of inflammation and fibrosis was also observed in the intestines of mice of the SnPP + DHA group than in those of the DHA-treated group (Figure 5E,F, Figure S5C and S5D). These results confirmed that the action of DHA could be partially reversed by HO-1 inhibition in both colitis models. 

### 2.6. DHA Regulates the Th/Treg Balance in OXA- and TNBS-Induced Colitis via HO-1

**Figure 6:**
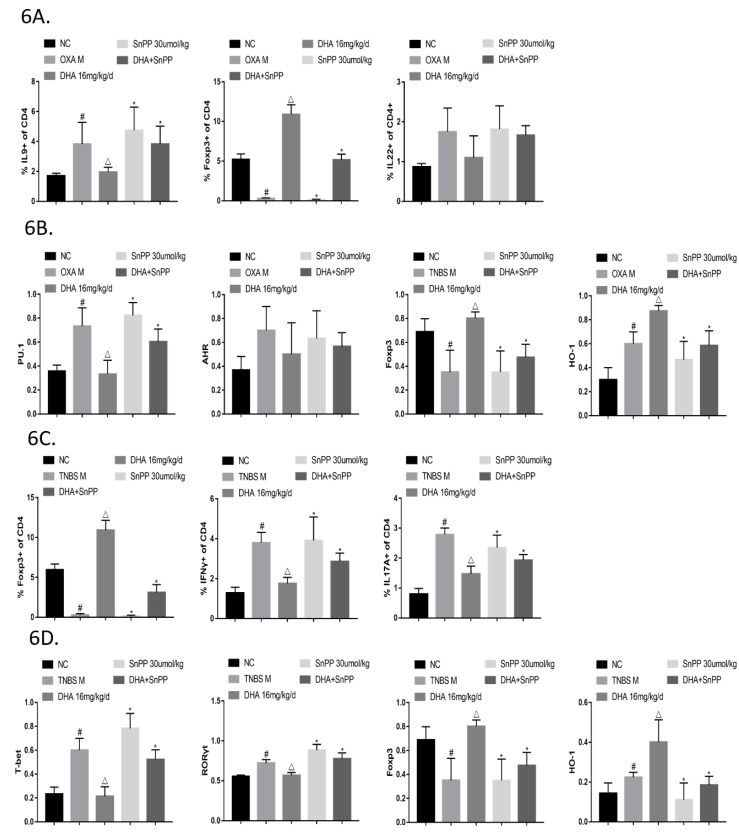
Dihydroartemisinin (DHA) regulates the Th/Treg balance in oxazolone (OXA)- and 2,4,6-trinitro-benzene sulfonic acid (TNBS)-induced colitis via HO-1. (**6A**) Lymphocytes were isolated from mouse spleens. CD4+IL9+, CD4+IL22+ and CD4+CD25+Foxp3+ cells were determined by flow cytometry. (*n* = 5). (**6B**) Lamina propria mononuclear cells (LPMCs) were isolated and subjected to RIPA lysis for protein collection. The protein amounts of PU.1, AHR, Foxp3, and HO-1 were estimated by capillary electrophoresis immunoblotting. (*n* = 3). (**6C**) Lymphocytes were isolated from mouse spleens. CD4+IFNγ+, CD4+IL17A+ and CD4+CD25+Foxp3+ cells were determined by flow cytometry. (*n* = 5). (**6D**) Lamina propria mononuclear cells (LPMCs) were isolated and subjected to RIPA lysis for protein collection. The protein amounts of T-bet, Foxp3, and HO-1 were estimated by capillary electrophoresis immunoblotting. (*n* = 3). (Compared to the NC group, #*p* <0.05, compared to the model group, △*p* <0.05, compared to the DHA group, *p <0.05).

Flow cytometric analysis of spleen lymphocytes of all groups was further performed to test whether SnPP could suppress the effect of DHA in regulating the Th/Treg balance. In the OXA colitis model, there was a greater proportion of Th9 cells and lower proportion of Treg cells in the SnPP+DHA group compared to those in the DHA-treated-group. Although Th22 cells tended to increase in the SnPP+DHA group, the difference was not statistically significant (Figure 6A). In the TNBS colitis model, SnPP significantly diminished the inhibitory effects of DHA on Th1 and Th17 cells and enhanced the effect of Treg cells (Figure 6C).

Similarly, the DHA-induced decrease in the PU.1 and AHR protein expression in LPMCs of mice with OXA-induced colitis was ameliorated, along with the decrease in T-bet and RORγt in TNBS colitis mice when HO-1 was inhibited (Figure 6B,D). Regarding Treg cells, the tendency for Foxp3 expression to be upregulated in the DHA group was also diminished upon treatment with SnPP (Figure 6B,D).

## 3. Discussion

Some artemisinin derivatives have been shown to have good efficacy for the treatment of TNBS- and dextran sulfate sodium-induced colitis [25,26]. However, the artemisinin derivative artesunate was also shown to aggravate OXA-induced colitis, and in this study, all of the artesunate-treated mice died, resulting in a higher mortality rate than that in the model group [27]. Here, we showed that DHA can improve body weight, clinical manifestations, and the survival rate in both OXA- and TNBS-induced colitis, suggesting that slight differences in chemical structures among artemisinin family members might greatly alter pharmacological effects. According to assessments of colon weight and length, DHA was also found to improve the colon injury and atrophy caused by the disease. DHA also reduced the local inflammatory response and tissue structure damage according to an evaluation of H&E-stained sections. Moreover, Sirus Red staining showed that DHA exerted potent effects with respect to the amelioration of tissue fibrosis, which is typically more difficult to treat than inflammation. Therefore, DHA has the potential to combat IBD, and the detailed mechanism is worthy of further exploration.

For IBD, previous research on the mechanisms underlying the activity of the artemisinin family found correlations with anti-inflammatory effects [25,26]. This study highlighted the interaction between DHA and the host immune system to explore its immunoregulatory potency. To evaluate each CD4+ T cell subset, lineage-specific transcription factors like Foxp3 for Tregs, T-bet for Th1 cells, and RORγt for Th17 cells were tested. Meanwhile, high expression of PU.1 for Th9 cells and AHR for Th22 cells were found to be important pathogenic regulators of IBD progression in recent years, yet therapeutic methods to target Th9 and Th22 cells are still preliminary. In this study, qRT-PCR was used to detect lineage-specific transcription factor and cytokine mRNA levels from different CD4+ T cell subsets. DHA was found to inhibit Th9 and Th22 cells in OXA-induced colitis and Th17 cells in TNBS-mediated colitis, and increased Tregs in both models. This is in agreement with the suppression of Th9 and Th22 transcription factors at the protein level in the OXA model and Th17 in the TNBS model. Further, western blotting results showed that Th1 cells were also significantly inhibited by DHA in the TNBS model. Simultaneously, DHA shifted the Th/Treg profile in spleen lymphocytes based on flow cytometric methods. There are many different types of anti-inflammatory or immunosuppressive agents in use, but the utilization of immunomodulators, which are even more desperately needed, is still in the initial stages. Herein, we propose that DHA has the potential to inhibit Th9 and Th22 cells, in addition to Th1 and Th17 cells, indicating that DHA might be able to effectively control the Th/Treg balance. Rather than global inhibition of the immune system, DHA appears to function more like an immunoregulator, which might be worth exploring for other autoimmune diseases related to Th/Treg disorders.

Before proliferating and differentiating into various subsets, which ultimately affects the Th/Treg profile during the primary immune response, T cells need to be activated by cognate antigens [28]. The over-activation of CD4+ T cells and resistance to apoptosis are hallmarks of autoimmune diseases and also mechanisms underlying Th/Treg imbalances [29,30]. Hence, the induction of activated CD4+ T cell apoptosis could be a feasible solution to maintain immune homeostasis. Various studies have shown conflicting results about the ability of artemisinin family members to suppress activated CD4+ T cells via apoptosis. Hou et al. [31] reported that SM934 could induce apoptosis in anti-CD3/28 activated CD4+ T cells, whereas Zhao et al. [10] indicated that DHA repressed anti-CD3/28 activated CD4+ T cells by inhibiting cell proliferation rather than inducing apoptosis. In the present study, we found that DHA decreased the number of anti-CD3/28-activated CD4+ T cells via apoptosis. Further, DHA decreased activated CD4+ T cell numbers in a dose-dependent manner in vitro, and decreased lamina propria cells in vivo. Based on Annexin V/PI double staining, DHA also induced activated CD4+ T cell apoptosis. The different results among studies are likely related to the drug dosage and the addition of IL-2. In particular, the dose of DHA was doubled in this study compared to that used in the previous study. Since a high dose (>1 µg/mL) of DHA is considered toxic to T cells, 0.8 µg/mL DHA was found to exert the best effects with respect to activated CD4+ T cell suppression in the other groups. In addition, we did not use IL-2 in this study since IL-2 signaling itself has been proposed as an attractive therapeutic target for autoimmune and T cell activation. Moreover, the results of TUNEL staining were consistent with those of Annexin V/PI double staining and confirmed that the inhibitory effect of DHA on activated CD4+ T cells was related to apoptosis. Instead of other ways to control activated CD4+ T cells like direct killing or global inhibition, inducing apoptosis might be one of the least costly options during the immunosuppression process.

HO-1 was previously shown to inhibit the accumulation of activated CD4+ T cells [32] and induce activated CD4+ T cell apoptosis through the Fas/CD95-FasL signal transduction pathway [33]. In this study, DHA accelerated HO-1 expression in vitro, which was accompanied by a reduction in CD4+ T cell numbers and the induction of activated CD4+ T cell apoptosis. Whereas HO-1 was downregulated by SnPP, activated CD4+ T cell numbers were significantly increased, and the proportion of apoptotic cells was significantly decreased. TUNEL staining results in vivo suggested the same trend. Therefore, DHA can enhance activated CD4+ T cell apoptosis via HO-1 induction.

HO-1 and its metabolic products CO were found to play critical roles in immune tolerance [34]. HO-1 can improve the pathologic outcome of EAE [35] and autoimmune hepatitis [36]. CORM-A1 (Carbon monoxide-releasing molecule-A1), which releases water-soluble CO, has been demonstrated to significantly ameliorate experimental autoimmune uveoretinitis [37], proteolipid protein-induced EAE [38], and type 1 diabetes [39]. Importantly, HO-1 activation reduces inflammation in the gut and has also been associated with colorectal cancer and intestinal bacterial [40], which might be a research hotspot in the near future. Nonetheless, HO-1 has not been studied with respect to the immunomodulatory effects of the artemisinin family. In this study, results showed that HO-1 is important for the ability of DHA to regulate immune homeostasis. Specifically, HO-1 was found to be upregulated by DHA in both OXA and TNBS models. Moreover, when HO-1 was inhibited by SnPP, the protein expression of Th cell lineage-specific transcription factors was increased and Treg cells were decreased. Meanwhile, the Th/Treg profile in spleen lymphocytes, estimated by flow cytometry, was changed in a similar manner. Hence, DHA regulates Th/Treg dysregulation through HO-1 induction.

In this study, the attenuation of OXA- and TNBS-induced colitis, mediated by DHA, was found to correlate with HO-1. The routine evaluation of disease-associated parameters in OXA- and TNBS-induced colitis after DHA-treatment, such as body weight, DAI, and survival rate, indicated that disease signs significantly worsened when HO-1 was suppressed by SnPP. For example, local lymphocyte infiltration and fibrosis were exacerbated in the SnPP+DHA group compared to those in the DHA group. This implies that the protective effect of DHA is mediated by HO-1 induction.

Additionally, macrophage migration inhibitory factor (MIF) is thought to play an important role in the pathogenesis of autoimmune diseases, especially in both rodent and human IBD [41]. MIF is also overexpressed in various malignant tumors such as brain tumors [42], breast cancer [43], and head and neck cancer [44]. Particularly, as chronic exposure to MIF has been shown to promote gastric and colon cancer, it might be an important link between chronic inflammation and tumorigenesis of the gastrointestinal tract [45]. Since artesunate has been shown to inhibit the production of MIF in patients with SLE [46], the effect of DHA on MIF expression during IBD will be explored in our future study.

In conclusion, DHA exerts an anti-colitis effect by regulating the Th/Treg balance, and the mechanism is closely related to the induction of activated CD4+ T cell apoptosis via the regulation of HO-1. Thus, DHA is a promising candidate for the treatment of IBD and other diseases related to Th/Treg dysregulation.

## 4. Materials and Methods

### 4.1. Colitis Induction and Disease Assessment

Colitis was induced in Balb/c mice (20–22 g, male, National Institutes for Food and Drug Control, Beijing, China). The mice were provided with standard mouse chow and water in a specific pathogen-free animal laboratory. All animal experiments were conducted under protocols approved by the Committee for Control and Supervision of Experiments on Animals of Institute of Chinese Materia Medica, China Academy of Chinese Medical Sciences. (No.2018-019)

The model mice received a single intracolonic application of TNBS or OXA, and the DAI was assessed daily as described previously [24]. In brief, the mice were pre-sensitized with a 3% OXA (Sigma-Aldrich, San Francisco, USA) or 1% TNBS (Sigma-Aldrich, USA) solution 7 days before rectal challenge. On the day of challenge, 0.1 mL of a 1% OXA or 2.5% TNBS solution was slowly instilled via a catheter positioned at least 4 cm proximal to the anus. The mice were maintained in a vertical position for 1 min, and then returned to their cages. Paired weight-matched negative control mice underwent the same procedure, except they were administered normal saline. The DAI was assessed according to weight loss, stool consistency, and the degree of intestinal bleeding (Table 1).

### 4.2. In Vivo Administration of DHA or SnPP

DHA (Chongqing Holley Wuling Mountain Pharmaceutical Company, purity ≥ 99%) was dissolved in corn oil. At 8, 24, and 48 h after OXA or TNBS infusion, the mice were administered 4, 8, or 16 mg/kg DHA per day or 1 mg/kg dexamethasone (DXM) per day intraperitoneally three times. In the control and model groups, the mice were administered the same volumes of corn oil. The corn oil, provided by Jiangxi Yipusheng Pharmaceutical Co., was at the pharmaceutic adjuvant level, conforming to the standards of the “Pharmacopoeia of China” (edited in 2015).

To assess the effects of HO-1 inhibition, the mice were administered 30 μmol/kg of SnPP subcutaneously into the posterior neck tissue at 0, 24, and 48 h after infusion three times, whereas the normal control and model groups were administered normal saline. All mice were sacrificed by cervical dislocation 72 h after infusion.

### 4.3. Histopathology

The entire colons from the OXA groups and the entire intestines from the TNBS groups were quickly removed and cleaned with ice-cold saline. The colons and intestines were fixed with 4% paraformaldehyde and embedded in paraffin. Tissue sections (3-µm-thick) were stained with H&E and Sirius-Red, and the degree of inflammation was observed under a microscope and scored from 0 to 4 as follows: 0, no signs of inflammation; 1, low leukocyte infiltration; 2, moderate leukocyte infiltration; 3, high leukocyte infiltration, moderate fibrosis, high vascular density, thickening of the colon wall, moderate goblet cell loss, and focal loss of crypts; and 4, transmural infiltration, massive loss of goblet cells, extensive fibrosis, and diffuse loss of crypts [47]. The percentages of positive collagen fiber areas in the colon or intestine were calculated using Image-pro plus 6.0.

### 4.4. Preparation of Spleen Lymphocytes and LPMCs

The spleens were removed from the mice, passed through a 100-μm cell strainer, flushed with PBS buffer, centrifuged, and resuspended in a 30–70% Percoll solution (GE Healthcare, PittsburghUSA) density gradient. After centrifugation at 550× *g*, the lymphocytes in the middle layer were collected.

LPMCs were extracted according to a previously reported modified method [48]. The harvested colons or intestines were washed with complete HBSS without Ca^2+^ and Mg^2+^, cut into 5-mm pieces, and incubated in medium containing 5 mM EDTA (Sigma-Aldrich, USA) and 1 mM DTT (Sigma-Aldrich) at 37 °C for 20 min twice until all crypts and individual epithelial cells were removed. The samples were then further incubated with complete HBSS without Ca^2+^ and Mg^2+^ at 37 °C for 20 min to remove any remaining EDTA and DTT. The tissues were digested in complete HBSS with Ca^2+^ and Mg^2+^ containing 5% fetal calf serum (Gibco, New York, USA), 0.15 g of collagenase type IV (Sigma-Aldrich), and 0.025 g of DNase I (Sigma-Aldrich) in a shaking incubator at 37 °C for 30 min. The tissue slurry was then passed through a 100-μm cell strainer to remove undigested tissue pieces, centrifuged, and resuspended in a 30–70% Percoll solution (GE Healthcare, USA) density gradient. After a final centrifugation at 550× *g*, the lymphocytes in the middle layer were collected.

The spleen cells were treated with DHA dissolved in DMSO (Sigma Aldrich) for 72 h before further procedures. In addition, the cells were treated with the HO-1 inhibitor SnPP (Santa Cruz Biotechnology, *Santa* *Cruz*, USA), dissolved in 0.1 mL of 0.5 M NaOH adjusted to pH 7.4 with PBS buffer.

### 4.5. Flow Cytometry

Cells were stained with fluorescence-conjugated antibodies for markers of Tregs and Th cells, including CD4, IFN-γ, IL-9, IL-17, IL-22, and Foxp3 (BD Pharmingen), according to the manufacturer’s protocols. For cytokine staining, the cells were stimulated using a Leukocyte Activation Cocktail with BD GolgiPlug™ for 6 h at 37 °C with 5% CO_2_. For intranuclear staining, the cells were mixed with the fixation/permeabilization working solution and incubated for 30 min. Flow cytometric analysis of antibody-labeled cells was performed on a BD Accuri C6 system. The details were provided in the supplementary material. The numbers of cell subpopulations were calculated by multiplying the total cell number by the percentages of each subpopulation, as determined by flow cytometry.

### 4.6. Quantitative RT-PCR

Total RNA from the colon or intestine tissues was isolated using TRIzol reagent according to the manufacturer’s instructions. The cDNA was transcribed from RNA using PrimeScript RT Reagent Kit (Takara Bio, Beijing, China), which was used to determine the relative expression levels of *GADPH*, *T-bet*, *PU.1*, *RORγt*, *AHR*, *Foxp3*, *IFN-γ*, *IL-9*, *IL-22*, and *IL-10* with qPCR SYBR Green Master Mix (Transgen, Beijing, China) on a qTower3/G system (Analytik Jena AG, Turingia, Germany). The mRNA expression level of each gene was normalized to levels of the reference gene, GADPH, and expressed relative to the normal control group according to the 2−△△Ct method. All primers were synthesized by Beijing Ruiboxingke Biotech Company (Beijing, China). Primer sequence were provided in the supplementary material. 

### 4.7. T Cell Apoptosis Assays

Flow cytometric and TUNEL assays were respectively used to determine the potential influence of DHA on T cell apoptosis. For flow cytometry, CD4+ T cells purified with MACS beads (Miltenyi Biotec) were stimulated with plate-bound anti-CD3e and anti-CD28 for 72 h, and then stained with Annexin V-FITC and PI according to the manufacturer’s protocols (Absin, Shanghai, China) and analyzed by flow cytometry.

For TUNEL staining, slides were stained with the reagents supplied by the TUNEL Kit (Roche, Switzerland). All TUNEL-positive cells assessed in three different unit areas were counted. The average number of cells per unit area number for each set of specimens in each group was calculated and compared.

### 4.8. ELISA and Immunoblotting

The concentrations of protein lysates from LPMCs were determined by a BCA assay. Equal concentrations of protein lysates of all samples were detected using an Automatic Wes system (ProteinSimple Biosciences & Technology, Shanghai, China) with the capillary electrophoresis western blotting method. The detail of antibodies were provided in the supplementary material. 

The concentrations of HO-1 in the cell culture media were measured by ELISA according to the manufacturer’s instructions (Abcam, Cambridge, UK).

### 4.9. Statistical Analysis

All values are expressed as means ± standard deviations. Repeated measures like body weight index and DAI were assessed by a two-way ANOVA. Data were compared among groups using a one-way ANOVA. Values of p <0.05 were considered statistically significant.

## Figures and Tables

**Table 1:** Scoring system to calculate disease activity index based on weight loss, stool consistency, and the degree of intestinal bleeding.

Score	Weight Loss	Stool Consistency	Blood
0	None	Normal	Negative hemoculture
1	1–5%	Soft but still formed	Negative hemoculture
2	6–10%	Soft	Positive hemoculture
3	11–18%	Very soft; wet	Blood traces in stool visible
4	>18%	Watery diarrhea	Gross rectal bleeding

## Supplementary Materials

The following are available online, Figure S1: Dihydroartemisinin (DHA) ameliorates oxazolone (OXA)-induced colitis in a dose-dependent manner in vivo, Figure S2: Dihydroartemisinin (DHA) ameliorates 2,4,6-trinitro-benzene sulfonic acid (TNBS)-induced colitis in a dose-dependent manner in vivo, Figure S3: Dihydroartemisinin (DHA) regulates the Th/Treg balance in oxazolone (OXA)- and 2,4,6-trinitro-benzene sulfonic acid (TNBS)-induced colitis, Figure S4: DHA Suppresses Activated CD4+ T Cell Subsets by Inducing Apoptosis via HO-1, Figure S5: Dihydroartemisinin (DHA) ameliorates oxazolone (OXA)- and 2,4,6-trinitro-benzene sulfonic acid (TNBS)-induced colitis via HO-1.
