# Supplementary material for: Dihydroartemisinin Regulates the Th/Treg Balance by Inducing Activated CD4+ T cell Apoptosis via Heme Oxygenase-1 Induction in Mouse Models of Inflammatory Bowel Disease
Source: Molecules. 2019 Jul 5;24(13):2475. doi: 10.3390/molecules24132475 (PMC6651826; doi:10.3390/molecules24132475)
Supplement: Supplementary file 1 [file molecules-24-02475-s001.pdf]

1. Supplemantory method

1.1 Quantitative RT-PCR primer sequences were as follows: GAPDH forward 5′-GGTGAAGGTCGGTGTGAACG-3′ and reverse 5′-CTCGCTCCTGGAAGATGGTG-3′; T-bet forward 5′- AGCCAGCCAAACAGAGAAGACTCA-3′ and reverse 5′- AATGTGCACCCTTCAAACCCTTCC-3′; PU.1 forward 5′-AGGAGTCTTCTACGACCTGGA-3′ and reverse 5′-GAAGGCTTCATAGGGAGCGAT-3′; RORγt forward 5′-GTGGACTTCGTTTGAGGAAAC-3′ and reverse 5′-ACTTCCTCTGGTAGCTGGTCAC-3′; AHR forward 5′-AGCCGGTGCAGAAAACAGTAA-3′ and reverse 5′-AGGCGGTCTAACTCTGTGTTC-3′; Foxp3 forward 5′-ATGCCCAACCCTAGGCCAGCCAAG-3′ and reverse 5′- TGGGCCCCACTTCGCAGGTCCCGAC-3′; IFN-γ forward 5′- CTGCTGATGGGAGGAGATGT-3′ and reverse 5′-TGTCATTCGGGTGTAGTCACA -3′; IL22 forward 5′-GTGAGAAGCTAACGTCCATC-3′ and reverse 5′- GTCTACCTCTGGTCTCATGG -3′; IL-10 forward 5′-CGGGAAGACAATAACTGCACCC-3′ and reverse 5′-CGGTTAGCAGTATGTTGTCCAGC-3′.

1.2 Flow cytometry assay details were as follows: For OXA-induced colitis, CD4-PerCP Cy5.5 (Channel 3), IL9-PE (Channel 2), and IL22-Alexa Fluor® 647 (Channel 4), as well as CD4-PerCP Cy5.5 (Channel 3), CD25-Alexa 647 (Channel 4), and Foxp3-PE (Channel 2), were used. For TNBS-induced colitis, CD4-APC (Channel 4), IL17-PE (Channel 2), and IFNγ-FITC (Channel 1), as well as CD4-PerCP Cy5.5 (Channel 3), CD25-Alexa 647 (Channel 4), and Foxp3-PE (Channel 2), were used.

1.3 Antibody details used in the capillary electrophoresis western blotting method were as follows: Antibodies against T-bet (Proteintech, China), PU.1 (Cell Signaling Technology, USA), RORγt (Abcam, Cambridge, US), AHR (Proteintech, China), HO-1 (Cell Signaling Technology, USA), and β-actin (Abcam, Cambridge, USA) were used for immunoblotting as per the manufacturer's protocols.

2. Supplemantory result

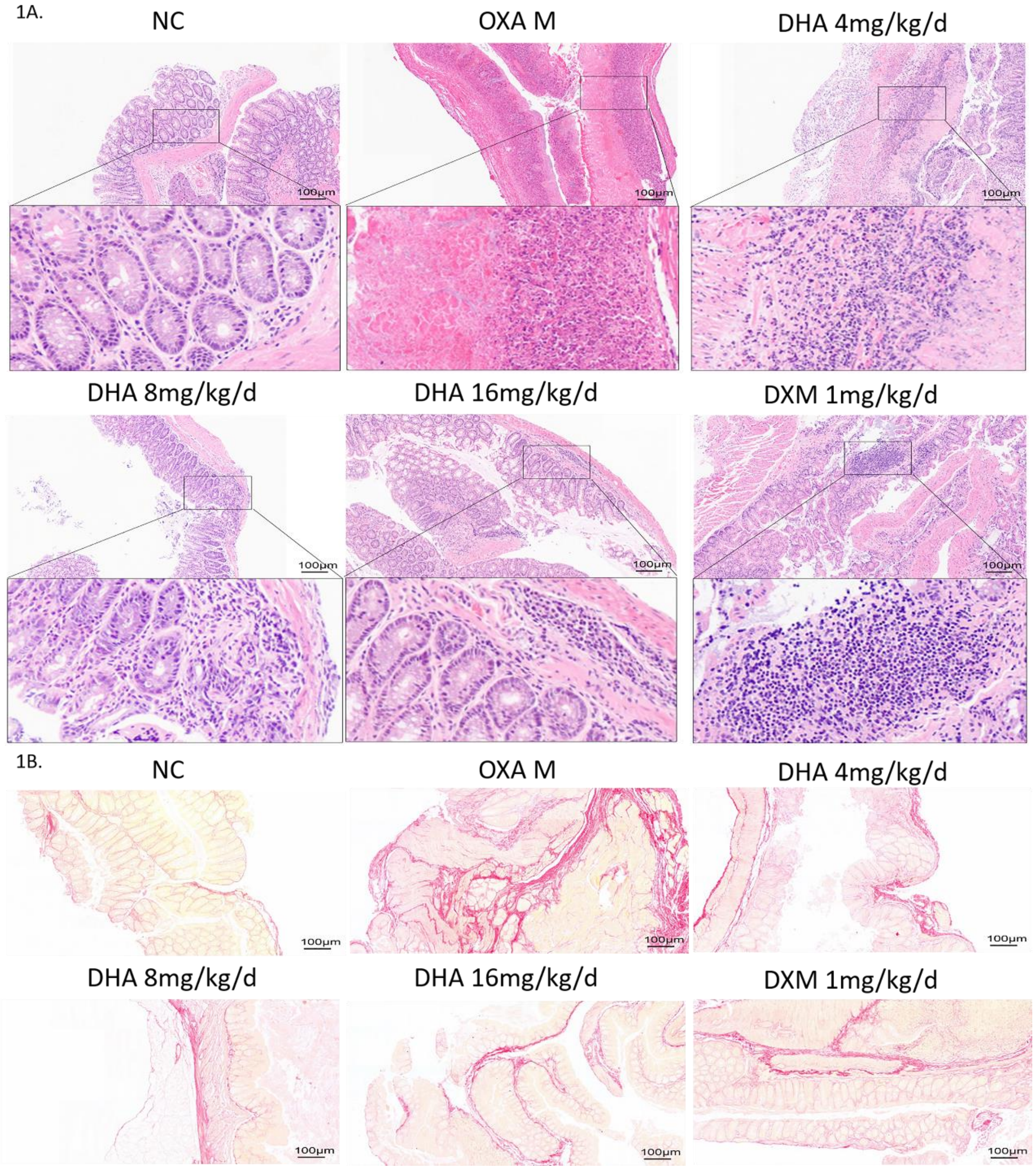

Supplementary Figure 1. Dihydroartemisinin (DHA) ameliorates oxazolone (OXA)-induced colitis in a dose-dependent manner in vivo. (**1A**) As described in **(Figure 1A**), mice were administered OXA and treated with corn oil, DHA, or DXM. Typical images of colon H&E (Hematoxylin-Eosin) staining in each group are shown. (**1B**) As described in **(Figure 1A**), mice were administered OXA and treated with corn oil, DHA, or DXM. Typical images of Sirius Red staining of colons in each group are shown.

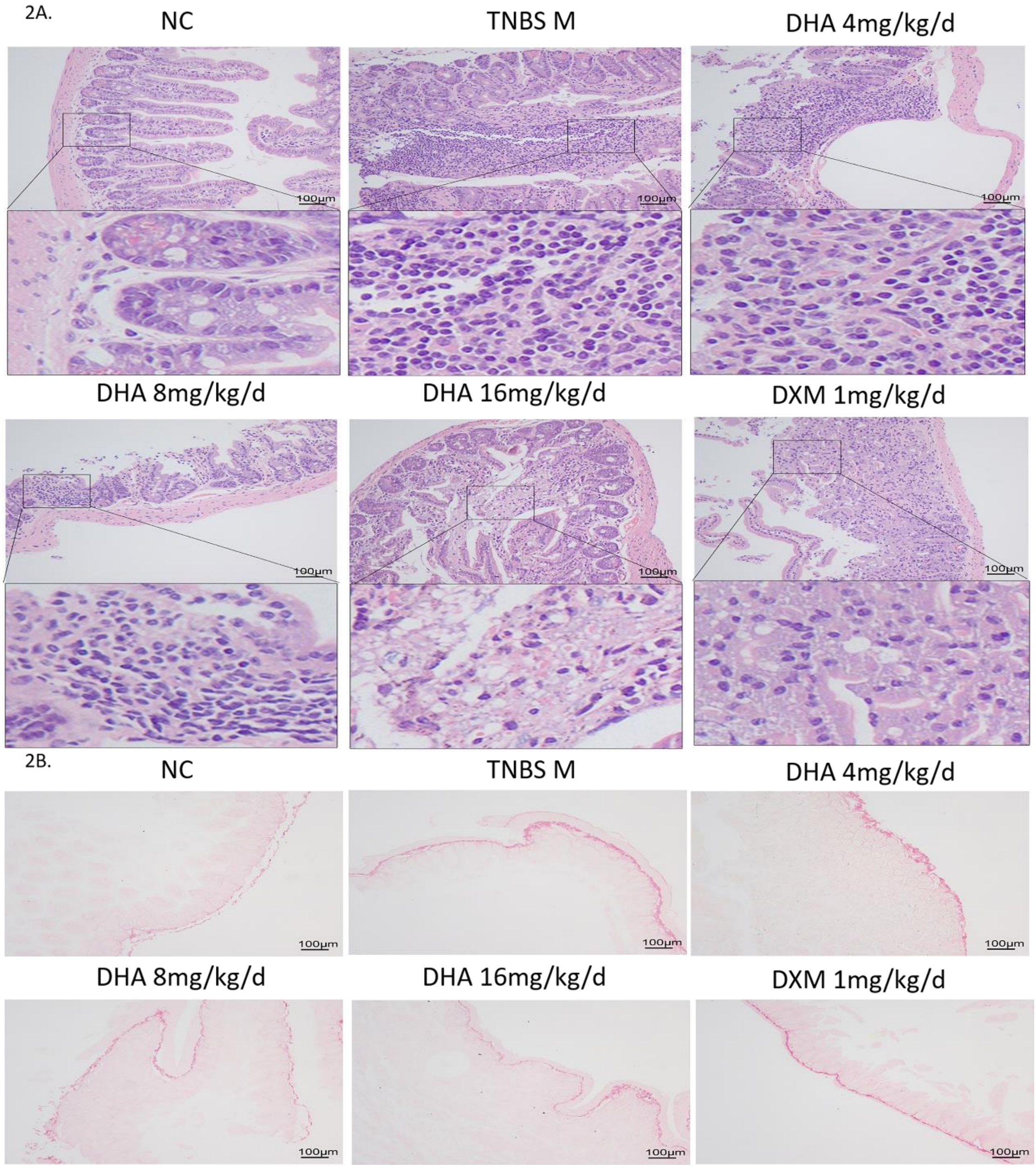

Supplementary Figure 2. Dihydroartemisinin (DHA) ameliorates 2,4,6-trinitro-benzene sulfonic acid (TNBS)-induced colitis in a dose-dependent manner in vivo. (**2A**) As described in **(Figure 2A**), mice were administered TNBS and treated with corn oil, DHA, or DXM. Typical images of colon H&E staining in each group are shown. (**2B**) As described in **(Figure 2A**), mice were administered TNBS and treated with corn oil, DHA, or DXM. Typical images of colon Sirius Red staining in each group are shown.

3A.

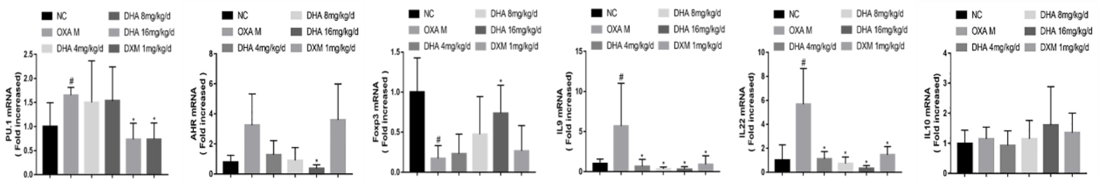

3B.

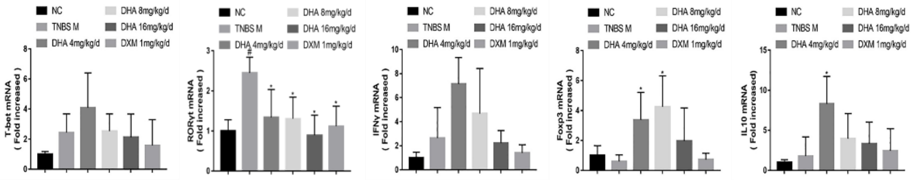

Supplementary Figure 3. Dihydroartemisinin (DHA) regulates the Th/Treg balance in oxazolone (OXA)- and 2,4,6-trinitro-benzene sulfonic acid (TNBS)-induced colitis. (**3A**) Colon tissue removed from the NC, OXA M, and DHA (4, 8, and 16 mg/kg/day) groups was used for mRNA extraction. The mRNA levels of *PU.1*, *AHR*, *Foxp3*, *IL9*, and *IL22* were detected by qRT-PCR (n = 4–6, compared to the NC group, #p <0.05, compared to the model group, *p <0.05). (**3B**) Intestine tissue removed from NC, TNBS M, and DHA (4, 8, and 16 mg/kg/day) groups was used for mRNA extraction. The mRNA levels of *T-bet*, *RORγt*, *Foxp3*, *IFNγ*, *IL17A*, and *IL10* were detected by qRT-PCR (n = 4–6, compared to the NC group, #p <0.05, the compared to the model group, *p <0.05).

4A.

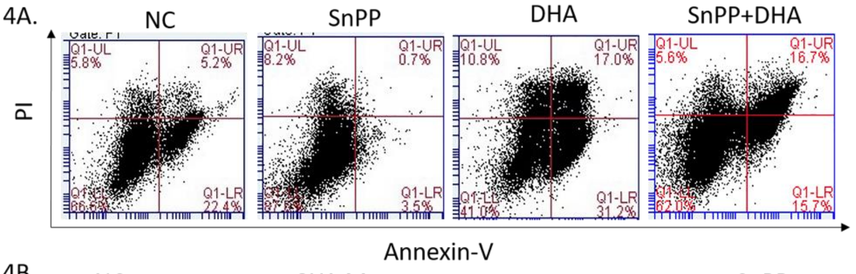

4B.

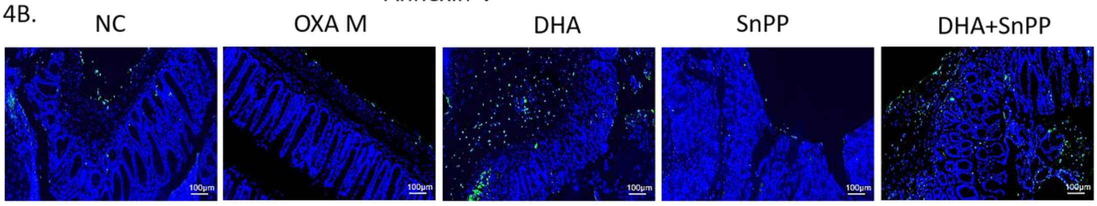

4C.

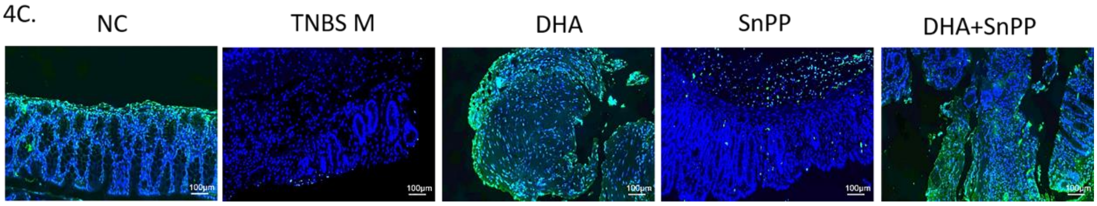

Supplementary Figure 4. DHA Suppresses Activated CD4+ T Cell Subsets by Inducing Apoptosis via HO-1. (**4A**) As described in **(Figure 4B**), remaining cells were stained with Annexin-V and propidium iodide (PI). The percentages of each population were detected by flow cytometry. Typical images are shown. (**4B)** Colons removed from NC and OXA mice were stained with TUNEL. The percentages of positive cells were calculated in each group. Typical images are shown. (**4C**) Intestines from NC and TNBS mice were stained with TUNEL. The percentages of positive cells were calculated in each

group. Typical images are shown.

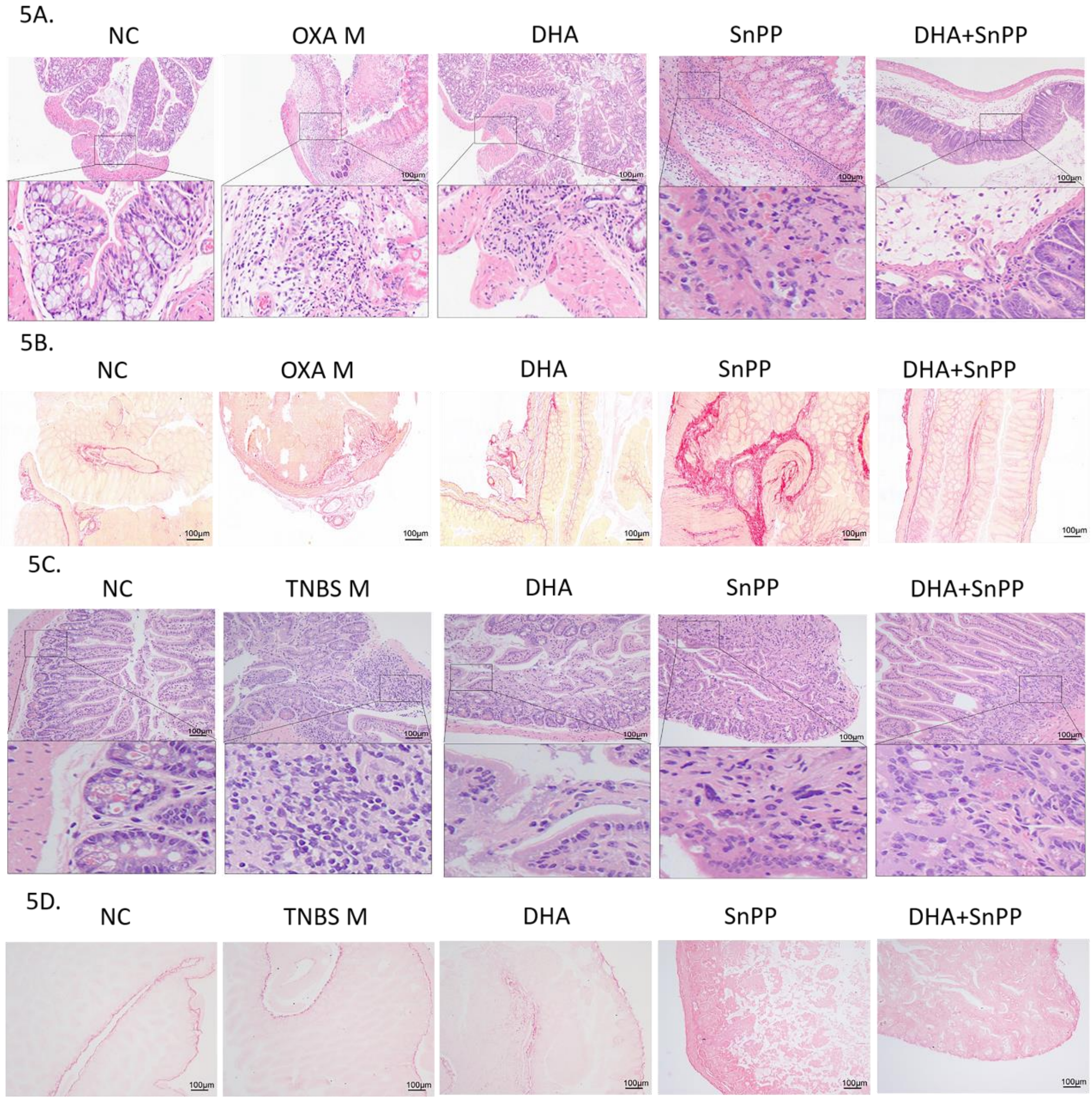

Supplementary Figure 5. Dihydroartemisinin (DHA) ameliorates oxazolone (OXA)- and 2,4,6-trinitro-benzene sulfonic acid (TNBS)-induced colitis via HO-1. (**5A**) As described in (Figure **5A**), mice were administered OXA and treated with corn oil, DHA, or SnPP. Typical images of colon H&E staining in each group are shown. (**5B**) As described in **(Figure 5A**), Typical images of Sirius-Red-stained colons in each group and percentages of collagen fibers area are shown. (**5C**) As described in (Figure **5D**), mice were administered OXA and treated with corn oil, DHA, or SnPP. Typical images of colon H&E staining in each group are shown. (**5D**) As described in **(Figure 5D**), Typical images of Sirius-Red-stained colons in each group and percentages of collagen fibers area are shown.
